# Backscatter-Assisted Collision-Resilient LoRa Transmission

**DOI:** 10.3390/s22124471

**Published:** 2022-06-13

**Authors:** Fei Xiao, Wei Kuang, Huixin Dong, Yiyuan Wang

**Affiliations:** 1 School of Navigation, Wuhan University of Technology, Wuhan 430074, China; feixiao@hust.edu.cn; 2 School of Management, Huazhong University of Science and Technology, Wuhan 430074, China; 3 School of Electronic Information and Communications, Huazhong University of Science and Technology, Wuhan 430074, China; huixin@hust.edu.cn (H.D.); yiyuanwang33@gmail.com (Y.W.); 4 TP-Link Technologies Co., Ltd., Shenzhen 518000, China

**Keywords:** LoRa, backscatter, collision elimination

## Abstract

Low-power wide-area networks (LPWANs), such as LoRaWAN, play an essential role and are expanding quickly in miscellaneous intelligent applications. However, the collision problem is also expanding significantly with the mass promotion of LPWAN nodes and providing collision-resilient techniques that are urgently needed for these applications. This paper proposes BackLoRa, a lightweight method that enables collision-resilient LoRa transmission with extra propagation information provided by backscatter tags. BackLoRa uses several backscatter tags to create multipath propagation features related to the LoRa nodes’ positions and offers a lightweight algorithm to extract the feature and correctly distinguish each LoRa node. Further, BackLoRa proposes a quick-phase acquisition algorithm with low time complexity that can carry out the iterative recovery of symbols for robust signal reconstructions in low-SNR conditions. Finally, comprehensive experiments were conducted in this study to evaluate the performance of BackLoRa systems. The experimental results show th compared with the existing scheme, our scheme can reduce the symbol error rate from 65.3% to 5.5% on average and improve throughput by 15× when SNR is −20 dB.

## 1. Introduction

Recent years have witnessed a massive promotion of low-power wide-area networks (LPWANs) such as LoRa, NB-IoT, and SigFox in real-time intelligent systems, such as the Intelligent Transportation System (ITS) [1,2,3,4]. These technologies enable intelligent transportation applications such as telemetries with autonomous vehicles, cooperative transportation networks, and smart roads. LoRa Wide Area Network (LoRaWAN), one of the most widely used LPWAN technologies that operate in the ISM band, is known for long-range, low-cost communication performance, creating heterogeneous connectivity, and achieving low-latency applications in high-capacity environments [3]. However, with the explosion of ITS deployments, collisions are increasingly becoming a significant problem, hindering the development of various intelligent applications based on LoRaWAN. Unfortunately, existing LoRaWANs that adopt the ALOHA *Media Access Control* (MAC) mechanism may further aggravate the collision when LoRa nodes are densely deployed. The Binary Exponential Back-off (BEB) scheme the ALOHA used even decreases channel utilization in networks with high real-time requirements [5,6], which is unaffordable by real-time networks such as ITS.

Existing physical-layer approaches [7,8,9,10,11,12,13,14,15] focus on adding the time of frequency offset to avoid collisions. However, it is impractical to accurately extract these small offsets because the LoRa signal strength is lower than the noise floor. Meanwhile, existing approaches [16,17,18,19,20,21] to the MAC layer attempt to reduce the collision through congestion control with different spreading factor (SF) allocations. Though these strategies can reduce the collision to some extent, LoRa nodes must make trade-offs between distance and data rate power consumption. The throughput improvement is thereby limited. Although these schemes work well in the cases of high signal-to-noise ratio (SNR), they are ineffective in the long-distance and wide-range operating contexts of LoRa signals, which suffer from low SNR. Therefore, a collision cancellation strategy for low-SNR environments is urgently needed.

We can observe that the fundamental obstacle in realizing a meager SNR collision resolution lies in the random segmentation of LoRa packets, which reduces the SNR with the division of LoRa symbols. The proportion of the divided parts is likely to be unbalanced and easily submerged by noise, which makes the SNR even lower. However, without packet detection and segmentation, it is extremely limited to take perceive and reduce collisions.

**Figure 1:**
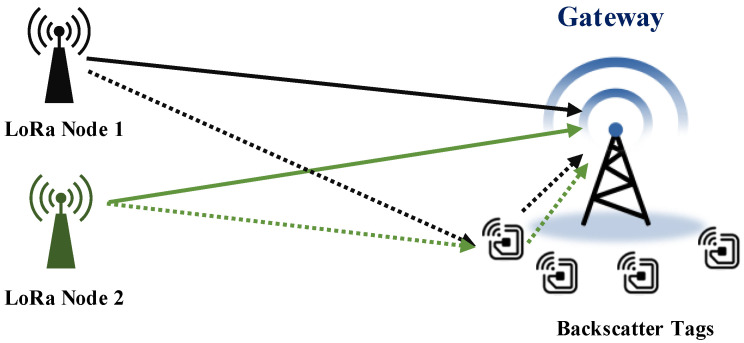
BackLoRa deployment. Battery-free backscatter tags are attached to the gateway to create multipath profile for LoRa node.

In this paper, we present BackLoRa, as shown in Figure 1, a lightweight system that uses independent backscatter tags to enable collision-resilient LoRa transmission. Our crucial insight is that these LoRa nodes are allocated in different positions with diverse distances to the receiver. If we accurately distinguish the location from the received LoRa signals, the colliding signals can be easily separated. Nevertheless, position estimation is not painless, and existing methods tend to receive and analyze signals’ *Angle of Arrival* (AoA) and *Time of Flight* (ToF) through multiple antenna systems. However, low-power, low-cost, and widely deployed LoRa gateways cannot afford the hardware cost and power budget for AoA and ToF computation. Fortunately, a new, low-power communication technology, backscattering [22,23,24], offers us a unique opportunity to distinguish the position of each LoRa node in a lightweight technical route. Inspired by previous outstanding work [25,26,27], we propose that using backscatter tags can intentionally create fine-grained position signatures by scattering ambient LoRa signals. Thus, we can extract the multipath information of each chirp on the receiver, distinguish the LoRa packets from different LoRa nodes, and further achieve collision-resilient LoRa transmission.

To realize the BackLoRa system, we encounter the following three challenges.

- *The accurate measurements and extraction of collide signals.* The demodulation of LoRa symbols relies on the accurate extraction of the peak amplitude in the Fast Fourier Transform (FFT) spectral sequence [2]. Furthermore, inevitably, the imprecise hardware clock of these low-cost backscatter tags causes the signal energy to be distributed across the spectrum, manifested as side lobes around the accurate peaks. Thus, eliminating the side lobes and accurately estimating the peaks are fundamental challenges in implementing collision-resilient LoRa transmission with backscatter assist.
- *Robust signal extraction.* Backscatter tags create propagation features in the collision with a lightweight method. Furthermore, it is challenging to distinguish different nodes and independently extract the signal from each node.
- *Reliable signature construction and recognition.* We require a lightweight method to use the position feature of each node, reliable signature construction, and recognition use of the signatures extracted from the collision to determine the whether the signals belong. However, it is challenging to map thousands of signatures when the LoRa nodes are densely deployed.

To enable a practical backscatter-based collision eliminating system, we prototype the BackLoRa system with the co-design of algorithm and hardware. Specifically, we provide a hardware design with harmonic frequency elimination and an accurate peaks extraction algorithm to remove the distortion and suppress the side lobs involved with the backscatter tags. We also offer a reliable signature construct scheme using backscatter tags and a recognition algorithm to determine whether the signals’ for accurate collision elimination. To date, we have prototyped BackLoRa tags with low-cost components and built up a test bench includinga commercial LoRa transceiver SEMTECH SX1276MBLAS for LoRa packet generation, USRP X310 with a daughterboard, model UBX160 for the receiver, and software version Labview 2014 for data recording. We have conducted comparative evaluation experiments in the real-world environment, and the results show that BackLoRa provides competitive performance in collision elimination.

The contributions of this paper are summarized as follows. We (i) propose BackLoRa using the position signature created by backscatter tags for collision elimination; (ii) develop a lightweight signature recognition algorithm to extract the signals that have collided, eliminate the collision, and reconstruct the signals further; and (iii) implement the system and conduct extensive experiments to evaluate the prototype.

The rest of this paper is organized as follows. Section 3 provides our key insight on using backscatter tags to create position signatures on LoRa signals and explains the feature of these signals theoretically. Section 4, Section 5, and Section 6 provide the design, implementation, and experimental evaluation of our system, respectively.

## 2. Related Work

Prior remarkable achievements on LoRa collision elimination [28,29] cleverly exploit the unique time-frequency characteristics of LoRa signals, converting temporal collisions into frequency characteristics. CoLoRa [28] translates time offset to frequency features for reliable collision cancellation. Nscale [29] also modulates the subtle time offsets to robust frequency features and further amplifies the time offset using non-stationary signal scaling. Unlike these approaches, BackLoRa utilizes the position features for collision cancellation with backscatter tags.

Multiple access technologies in cellular [7,8] and backscatter [9,10,11,13,14,30,31] networks focus on concurrent communications and are similar to packet recovery for collisions, which has inspired BackLoRa to distinguish signals with position features for independent collision cancellations. A proactive synchronization for concurrency is required for these approaches. However, it is difficult to implement collision cancellation in practice, as a collision can occur at any time when these packets are definitely out of synchronization.

## 3. Characterizing LoRa Packet Collision

Packet collisions prevent LoRaWAN from reaching a higher throughput and make it difficult to widely use in intelligent transportation applications. Before we provide the system design, it is worth noting the motivation and background to better understand our core idea. We first provide the feature of LoRa signals and argue that a lightweight mechanism designed for collision elimination is critical. Then, we describe the influence that the backscatter tags bring and provide methods to distinguish these features. Finally, we explain how to eliminate the impact theoretically as the basis of our algorithm.

### 3.1. LoRa Packets Collision Primer

**Figure 2:**
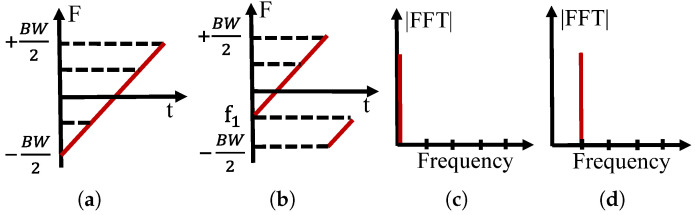
Illustration of LoRa modulation. (**a**) Base up-chirp. (**b**) An example of a modulated chirp symbol whose frequency start at $f_{1}$. (**c**) The demodulation result of (**a**). (**d**) The demodulation result of (**b**).

**Figure 3:**
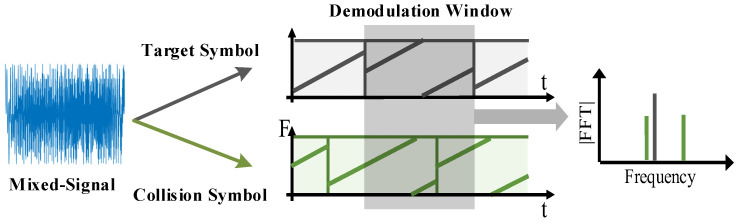
Example of LoRa collision.

Figure 2a,b show two kinds of LoRa symbols, and Figure 2c,d show the demodulation results. The LoRa protocol enables the transmission of information by modulating the start and end frequencies of the chirp signal and results in the change in the FFT bin after a multiplex with a down chirp signal. However, once the LoRa packets collide, as shown in Figure 3, there are be multiple FFT peaks after demodulation. The receiver needs to synchronize with one of the packets and align the demodulation window with each symbol in the packet for successfully decoding. Otherwise, the multiple peaks confuse the receivers in archiving these symbols to their packets, which will lead to demodulation errors. Existing approaches cannot proactively address this problem as an alternative to adopting a retreat approach. For example, ALOHA uses Binary Exponential Backoff (BEB) as a collision resolution [5], thus reducing the throughput in the network and limiting the applications of LoRaWAN. As shown in Figure 3, although the multiple peaks confuse the decoding process, the separate peaks in the demodulation process are measurable and contain the original message. If we successfully match these peaks to their original packets, it is promising to eliminate the collision and enable correct decoding. Thus, we can conclude that the critical point of decoding the packet is to pair the peaks in demodulating windows with their original packets.

### 3.2. Position Signature Creation with Backscatter Tags

**Figure 4:**
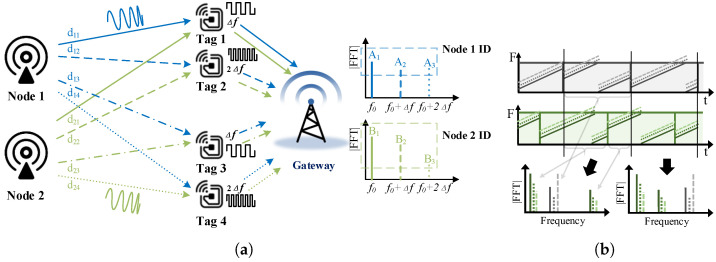
Signature creation with backscatter tags. (**a**) Position signature created by backscatter tags. (**b**) Collide packets with position signature.

A proactive approach that uses backscatter tags to create the signature is considered promising to eliminate the impact of collisions better. We deploy four backscatter tags to intentionally create the signature with backscatter tags around the gateway. Two of them operate with a frequency shift at Δf, and the other two tags operate with a frequency shift at 2Δf to enable multipath propagation in both $f_{c}+\Delta f$ and $f_{c}+2\Delta f$. Tags’ frequency shifts at Δf and 2Δf result in two additional peaks in the spectrum, as shown in the Figure 4a, and the multipath propagation created by backscatter tags makes the amplitude and phase sensitive to the LoRa nodes’ position.

The amplitude and phase of these additional signals are related to the distance between the LoRa nodes and the backscatter tags. We use the system model to explain how these tags work. As shown in Figure 4a, the peak $A_{1}$ represents the signal that comes directly from the LoRa node 1, $A_{2}$ is the signal from node1 that is scattered by $Tag_{1}$ and $Tag_{3}$, and $A_{3}$ is the signal from node1 that is scattered by $Tag_{2}$ and $Tag_{4}$. $B_{1}$, $B_{2}$, and $B_{2}$ are similar but for node 2. There are differences in two dimensions between $\left\{A_{1},A_{2},A_{3}\right\}$ and $\left\{B_{1},B_{2},B_{3}\right\}$ that can be used for classification and further collision elimination. One is that since the distances from these tags to node 1 are different from those to node 2, leading to the difference in the amplitude of these peaks and resulting in difference amplitude on average between $A_{1}$ and $B_{1}$. For the other, which is more important, the position difference of node1 and node2 leads to different multipath propagation gains, resulting in a significant difference between $A_{2}$ and $B_{2}$ and between $A_{3}$ and $B_{3}$. Figure 4b shows the collided packets, from which we can see the apparent signature created by backscatter tags, which also verifies the conclusion stated above. Thus, if we can deploy the backscatter tags properly, we can distinguish and extract the signal from each node with these features.

### 3.3. Collision Elimination with Scattered Signals

As concluded in Section 3.1, the key point of decoding the packet is to pair the peaks in demodulating windows with their original packets. We use the BackLoRa tags to create dynamic propagation features. The signals, which are obtained directly from the LoRa nodes and reflected by obstacles, cause static propagation that is constant for several packets. We can extract the signals propagated through BackLoRa tags by filtering out the static features, and we can also express the symbols in the signal as

(1) $$T\left(t\right)=\left\{\begin{array}{cc} e^{j2\pi (f_{0}+\frac{k}{2}t)t}, & t\in (0,T_{fold})\\ e^{j2\pi (f_{0}-BW+\frac{k}{2}t)t}, & t\in (T_{fold},T) \end{array}\right.$$

where $T_{fold}=T-\frac{f_{0}}{k}$ is the turning time of this symbol. The signal generated by the two tags with frequency shift $n*\frac{BW}{2^{SF}}$ Hz can be expressed as

(2) $$R_{1}\left(t\right)=H_{1}\left(t\right)\Gamma_{1}\left(t\right)T\left(t\right)$$

where $\Gamma_{1}\left(t\right)=Sgn\left(\sin\left(2\pi n\frac{BW}{2^{SF}}t\right)\right)$ represents the switching of the reflection coefficient. Furthermore, $H_{1}\left(t\right)$ is the path loss caused by signal propagation and multipath; if we denote the value of free-space path loss as α and carrier frequency as $f_{c}$, then $H_{1}\left(t\right)$ can be formulated as

(3) $$\begin{array}{cc} H_{1}\left(t\right) & =\alpha (e^{j2\pi f_{c}t}+e^{j2\pi f_{c}\left(t+\frac{\Delta d_{1}}{C}\right)})\\ \; & =\alpha e^{j2\pi f_{c}t}\left(1+e^{j2\pi f_{c}\frac{\Delta d_{1}}{C}}\right) \end{array}$$

where $\Delta d_{1}$ is the difference in the propagation path of the signals reflected by the two tags. The receiving gateway down-converts the received signal and performs the previously described LoRa demodulation method to obtain the result on the FFT spectrum as

(4) $$|R_{1}^{\prime }\left(f\right)|=|2\alpha \cos f_{c}\frac{\Delta d_{1}}{2c}F\left(f+n*\frac{BW}{2^{SF}}\right)|$$

where $F(f+n*\frac{BW}{2^{SF}})$ represents the FFT bin of the original LoRa symbol, shifted by *n* points. Furthermore, the amplitude of the the peak in the new FFT bin is related to Δd.

The symbols backscattered by two tags with frequency shift $2n*\frac{BW}{2^{SF}}$ Hz can be analyzed in the same way, with the peak in the original FFT bin shifted by 2n points and the peak amplitude related to the propagation difference $\Delta d_{2}$ between the signals. After receiving the mixed LoRa symbols, including one original symbol with two backscattered symbols, the receiver will show three peaks in the FFT spectrum, spaced *n* points from each other. The amplitude of each peak is related to the difference in propagation distance. In other words, each sender in a different place holds a unique amplitude signature. We name it *Symbol ID* to help the receiver distinguish their symbol from the collision signal and recover the packet. After we successfully extract and pair these *Symbol IDs* to their packets, it is easy to eliminate the collision with *Serial Interference Cancellation* (SIC) methods.

## 4. BackLoRa Design

When the collision occurs, the collision elimination algorithm starts with preamble detection and offset correction. Meanwhile, the backscatter tags begin to create propagation features by shifting the frequency of original signals by regularly switching, and each tag easily switches in a designed frequency for classification. Then, after the receiver acquires the signals, the BackLoRa system uses a symbol classification algorithm to extract the signal from each LoRa node with the features provided by backscatter tags. Finally, BackLoRa runs an iterative collision cancellation algorithm to reconstruct the original signals independently and demodulate the other signals. BackLoRa can detect the collided packets from unknown nodes and eliminate the collision in the same way. Once a packet is detected from an unknown node, BackLoRa starts to extract the propagation features created by BackLoRa tags from the collided packets and allocate the Symbol IDs to the new unknown nodes and then eliminates the collision in the same way described above.

**Figure 5:**
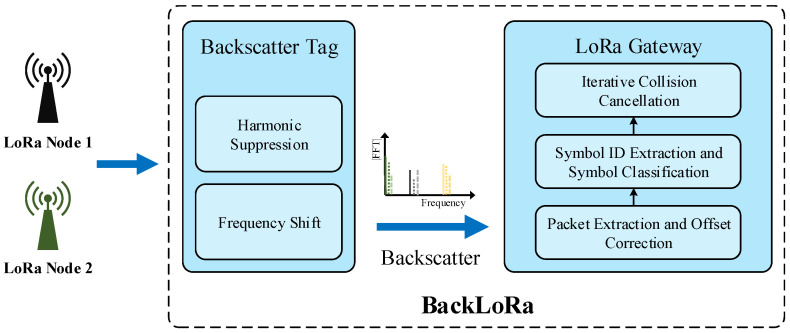
BackLoRa overview.

This section presents the details of BackLoRa, a new lightweight LoRa collision cancellation system that can be prototyped with off-the-shelf components. As shown in the Figure 5, BackLoRa consists of two parts. One consists of carefully designed backscatter tags that modulate propagation features on LoRa packets while keeping their energy in the μW level. The other is the collision elimination algorithm that can be deployed on a LoRa gateway.

### 4.1. Packet Detection and Synchronization

Packet detection and synchronization is the prime step for correct demodulation. However, the original approach of the LoRa system cannot be directly applied to the BackLoRa system because the backscatter tags create propagation signatures on the LoRa packet, which is regarded as interference for conventional LoRa receiver. Thus, making it challenging to detect and synchronize these LoRa packets correctly.

In this subsection, we propose and explain a packet detection and synchronization algorithm to conquer this challenge and enable the correct detection and synchronization in the BackLoRa system. The detection algorithm aims for sensitive preamble detection by applying SIC means on each acquired symbols, while the synchronization algorithm aims for accurate synchronizing by reducing the influence of *Carrier Frequency Offset* (CFO) and *Sample Frequency Offset* (SFO).

Specifically, the Algorithm 1 shows the detection process. We first find the starting point from the acquired signal in the time domain and then inter-correlate the received signals with standard up-chirp symbols. If a sequence of correlated peaks is spaced $2^{SF}$ from each other and repeated ten times, the same number of symbols in preamble, the LoRa packet is detected successfully. The first peak index is considered the start of the packet. Further, since the collide packet after scattering by BackLoRa tags contains multiple peaks in the spectrum, we begin the detection from the strongest signal and then the smaller one until all of the signals has been extracted.
**Algorithm 1** Packet Acquisition and Offset Cancellation. **Input: Raw Signal s(n)** **Output: Beginning of Packet $x_{begin}$, Corrected Signal $s^{\prime }\left(n\right)$** correlation(n) = Correlation( s(n),upchirp ); $correlation^{\prime }\left(n\right)$ = FalsePeaksRemove( correlation(n) ); **if** FindPreamble( correlation(n) ) is true **then**     $x_{begin}$ = GetPreamble( $correlation^{\prime }\left(n\right)$ );     $\delta_{downchirp}$ = FFTbins( s(SFDframe) );     $s^{\prime \prime }\left(n\right)$ = CoarseCorrection( $s\left(n\right),\delta_{downchirp}$ );     $s^{\prime }\left(n\right)$ = AccurateCorrection( $s^{\prime \prime }\left(n\right)$ ); **end if**

**Figure 6:**
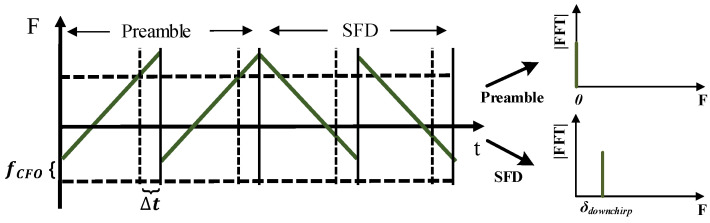
Example of carrier frequency offset and sampling time offset.

To enable the accurate packet synchronization obtained using the correlation peak, we need to eliminate the CFO and *Sampling Time Offset* (STO), as shown in Figure 6. Furthermore, these offsets result in changes in the FFT spectrum, which can be modeled by Equation (5)

(5) $$\begin{array}{cc} r(t) & =e^{j2\pi f_{CFO}t}T\left(t+\Delta t\right)\\ \; & =e^{j2\pi (f_{CFO}+k\Delta t)t}T\left(t\right) \end{array}$$

STO is the normalized offset and can be divided into an integer fraction and a fractional fraction. The integer fraction offset causes the FFT spectrum to shift by an integer number of points, while the fractional offset causes the energy to spread to the surrounding bins from the corresponding FFT bin.

The BackLoRa system is sensitive to the FFT peak amplitude and position. Thus, we leverage the down-chirp in the *Start Frame Delimiter* (SFD) after the preamble to correct the FFT spectrum and collect the diffused energy back to the corresponding bin. Specifically, positive STOs shift the spectral line in the same direction for up-chirps, but in opposite directions for down-chirps [32], which can be expressed as

(6) $$\left\{\begin{array}{c} \frac{2^{SF}}{BW}\left(f_{CFO}-k\Delta t\right)=0\\ \frac{2^{SF}}{BW}\left(f_{CFO}+k\Delta t\right)=\delta_{Downchirp} \end{array}\right.$$

where $\delta_{Downchirp}$ is the FFT bin obtained by demodulating SFD. We solve the equation and correct the signal coarsely.

To date, we correct integral multiples of the $\frac{BW}{2^{SF}}$ in frequency offset and $\frac{1}{BW}$ in time offset, remain fractional time, and frequency offset still exists, which results in the magnitude of the peak fluctuations in the FFT bin. Thus, we make use of the accurate offset estimation method mentioned in  [32] and successfully eliminate the effect of the fractional offset. In addition, considering the frequency shift caused by tags, three correlated peaks can be found in one symbol at coordinates [$n_{0}$–2n, $n_{0}$–n, $n_{0}$], which were created by the backscatter tags and should be ignored in order to select the correct ones and obtain the beginning of the packet.

### 4.2. Symbol Classification and Demodulation

Recall that in Section 3, we proposed a method using backscatter tags to modulate the position signature for each LoRa node, and the signature can be used to classify symbols in the demodulation window. Further, in Section 4.1, we extracted the original signal with accurate peak amplitudes measurement in the corresponding FFT bins. However, the packet collision causes multiple FFT peaks in the data segment decoding, and it is challenging to match the *Symbol ID* and these peaks. Moreover, as the channel state changes, the match process grows harder and makes it difficult to obtain and use *Symbol ID*, leading to a higher symbol error rate (SER).

To enable accurate *Symbol ID* matching, we propose a symbol classification and demodulation algorithm as shown in Algorithm 2. Specifically, we obtain the *Symbol IDs* and the index of these FFT bins from the preamble after synchronization. Then we use sliding windows to divide and match these packets. We group $2^{SF}$ FFT points within each window and measure peak amplitudes of every three points spaced by *n* points. Then, we calculate the *Euclidean* distance between these measured amplitudes and *Symbol IDs*, compare the *Euclidean* distance to determine the correspondence between measured peaks and collide packets, and realize the symbol classification further. We dynamically generate *Symbol IDs*, which co-exist with the changed wireless channels, with amplitude vectors $A_{i}$ and weights $w_{i}$ of five consecutive symbols.The following equation expresses the generation.

(7) $$A=w_{1}A_{1}+w_{2}A_{2}+w_{3}A_{3}+w_{4}A_{4}+w_{5}A_{5}$$

After the symbol is demodulated, we update the *Symbol ID* with measured amplitude to keep the algorithm robust in dynamic wireless channels.
**Algorithm 2** Symbol Classification and Demodulation. **Input: Symbol Frames in a Packet with Collision frame(x)** **Output: Symbol Value value** **while**frame(x) is in packets **do**     FFTbins = Demodulate( frame(x) );     **if** frame(x) is preamble **then**         SymbolID = CaculateSymbolID ( [FFTbins(1),FFTbins(1+N),FFTbins(1+2N)] );     **else**         **for** $i=1:2^{SF}$ **do**    FFTbinsDivide(i) = [ $FFTbins\left(i\right),FFTbins\left(mod\left(i+N,2^{SF}\right)\right),FFTbins\left(mod\left(i+2N,2^{SF}\right)\right)$];         **end for**         Symbol = SymbolWithMinmumDistance ( FFTbinsDivide,SymbolID );         value = FindLocation( Symbol );         SymbolID = CaculateSymbolID ( Symbol );     **end if**     frame(x)=frame(x+1); **end while**

### 4.3. Iterative Collision Cancellation

After we finish the classification and demodulation, collision cancellation is needed in order to successfully perform the decoding. However, collision cancellation is not easy because the spectrum of collision symbols contains complex mixed peaks, including peaks of target signals and peaks of harmonics caused by backscatter and side-lobes caused by incomplete symbols in other packets. The harmonic suppression design in [23] works but is limited and cannot handle the amplitude distortion problem when the energy of the target packet is lower than the collision packet. To enable the decoding in lower energy, we propose an iterative packet recovery algorithm, as shown in Algorithm 3. After a symbol is demodulated and decoded correctly, we reconstruct it with measured amplitude, phase, and initial frequency and then iteratively remove it from the original signal.

The details of our design for collision cancellation are as follows. Since the received signal consists of three parts, including the original symbols without frequency shifting, the frequency-shifted $n*\frac{BW}{2^{SF}}$ Hz copy, and the frequency-shifted $2n*\frac{BW}{2^{SF}}$ Hz copy, the received mixed signal can be expressed as

(8) $$r\left(t\right)=x_{1}e^{j\theta_{1}}T\left(t\right)+x_{2}e^{j\theta_{2}}T\left(t\right)e^{j2\pi n*\frac{BW}{2^{SF}}t}+x_{3}e^{j\theta_{3}}T\left(t\right)e^{j2\pi 2n*\frac{BW}{2^{SF}}t}$$

We start demodulation from the packet with the highest correlation peak in the preamble, which indicates that the packet has the highest power and can be less sensitive to colliding symbols. Next, we obtain the amplitude and phase from the revised FFT spectrum and the initial frequency from the FFT bin. Then, we can roughly reconstruct the symbol r(t). To obtain a more accurate estimate of the symbol, we slightly adjust the amplitude, phase, and initial frequency values for each frequency component separately and cancel it from the signal. The parameter’s value with the minimum signal energy left in the FFT spectrum is considered as that in the real symbol. Finally, the receiver removes it from the original signal and achieves the collision cancellation.
**Algorithm 3** Iterative Collision Cancellation. **Input: Symbol Frames in a Packet with Collision frame** **Output: Collision Canceled Signal $frame^{\prime }$** **while** 1 **do**     FFTbins = Demodulate( frame );     Symbol = FindSymbol( frame,SymbolID );     [$x1,x2,x3,\theta_{1},\theta_{2},\theta_{3},bin$] = GetInformation( Symbol );     r(n) = CoarseConstruct( $x1,x2,x3,\theta_{1},\theta_{2},\theta_{3},bin$ );     $r^{\prime }\left(n\right)$ = FurtherEstimation( r(n) );     $frame^{\prime }$ = frame−$r^{\prime }\left(n\right)$;     $FFTbins^{\prime }$ = Demodulate( $frame^{\prime }$ );     energy = Summarize( FFTbins );     **if** energy is minimum **then**         **return $frame^{\prime }$**     **end if** **end while**

## 5. Implementation

**Figure 7:**
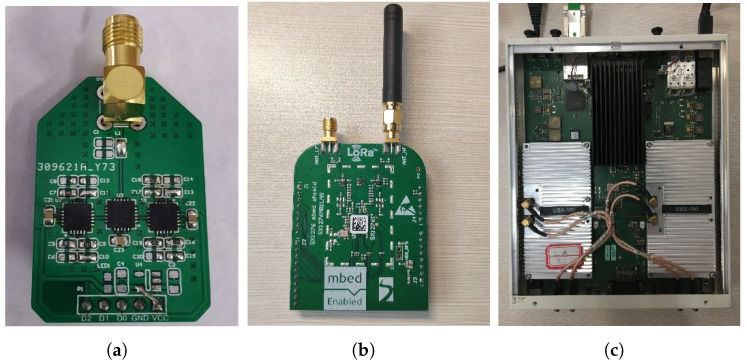
BackLoRa hardware and the test platform. (**a**) BackLoRa tag. (**b**) SX1276MBLAS LoRa transceiver. (**c**) USRP X310 with daughter board UBX160.

**BackLoRa tags.** It is worth noting that, since the backscatter tags we used to intentionally create propagation features are susceptible to harmonics, we designed and implemented a special backscatter tag with onboard harmonic elimination methods. Inspired by LoRa backscatter [23], we prototyped the tag with one pics of ADI ADG919 and two pics of ADI ADG904 to provide eight types of orthogonal impedance for harmonic elimination. We first conducted RF simulations to find the desired impedance and build the tag with RF switches. For fabrication, we used 1.6 mm FR-4 as the substrate, as shown in Figure 7a.

**Test platform.** We used the SEMTECH SX1276MBLAS LoRa EVM board as the LoRa transmitter node, and the USRP X310 with the UBX160 daughter board as the gateway to demodulate and record data in the 900 MHz band. The physical layer data received by USRP were input to MATLAB for data processing.

**Recognition algorithm.** To achieve the goal of collision elimination, we implemented a recognition algorithm running on Matlab version2020 software to process the origin signal. The PC was configured with a 64-bit Intel i7-10700 CPU.

## 6. Evaluation

In this section, we first introduce the experimental setup. Then, we evaluate the performance of our system with CoLoRa as the baseline. The results show that when the SNR is −20 dB, the throughput of our method increases steadily with the number of conflicting nodes. When the number of contradictory nodes increases to 20 dB, our throughput is 15× higher than that of CoLoRa. Further, We evaluate the throughput of our system in different SNRs with fixed tags. The results show that our approach also outperforms CoLoRa, and the superiority is more evident with the decreasing SNR. When the SNR is −20 dB, our solution reduces the false symbol rate from 65.3% to 5.5% on average compared to CoLoRa.

### 6.1. Experimental Setup

**Figure 8:**
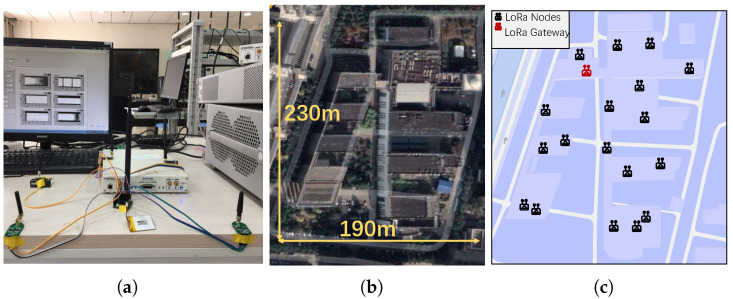
Experimental setup. (**a**) BackLoRa tags with USRP X310. (**b**) Experimental site map. (**c**) Floor plan with the location of gateways and nodes marked.

**Experimental environment.** We conducted the experiments in a five-floor open area with 190 × 230 $m^{2}$, as shown in Figure 8a. BackLoRa tags were deployed around an Ettus USRP X310 as plotted in Figure 8b. LoRa nodes were randomly placed in various locations such as corridors and rooms on the third floor of the building, and the USRP, as the gateway, was placed in the northwest corner of the third floor of the building, as shown in Figure 8c.

**Data-driven simulations.** Due to the limits of the experimental environment, conducting large-scale communication collision experiments is unrealistic, which requires all the decentralized deployment of Tag nodes to be well-synchronized or wait for a significant amount of time for the collision event to occur. Alternatively, we adopt the data-driven simulation approach to complete the evaluation of the system more efficiently. Specifically, we recorded the LoRa signals at every single location by moving several LoRa nodes after the Tag nodes are deployed, synthesized the collision signal on this basis, and then completed the verification of the system by performing a collision elimination algorithm.

**Baseline.** CoLoRa [28] and Nscale [29] are state-of-art approaches in achieving concurrent LoRa transmission with 3.4× and 3.3× throughput improvement in the low-SNR condition. Specifically, CoLoRa translates time offset to frequency features for reliable collision cancellation. Nscale modulates the subtle time offsets to robust frequency features and further amplifies the time offset by non-stationary signal scaling. Their performances are similar. We chose CoLoRa, which slightly outperformed NScale, as the baseline. To obtain the results of CoLoRa for comparison, we reproduced CoLoRa’s program according to the algorithm proposed in the paper and achieved a similar performance as the paper predicted. To ensure a fair comparison, we choose the same collide packets for CoLoRa and our system to eliminate the collision independently and then counted the number of successful collisions to evaluate the performance.

### 6.2. Classification Accuracy

**Figure 9:**
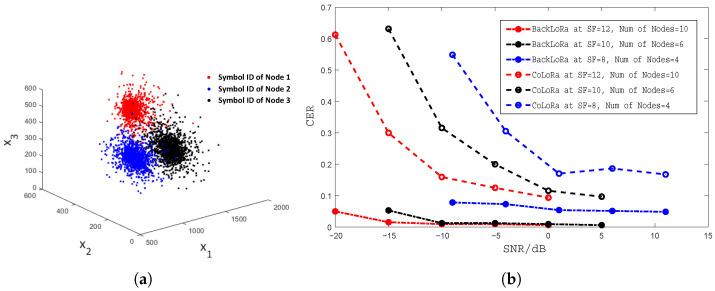
(**a**) *Symbol IDs* from different nodes at SNR from −20 dBm to 10 dBm. (**b**) Classification performance at different conditions compared with CoLoRa.

**Classification performance evaluation.** In order to evaluate the feasibility of our system, we deployed the test platforms and then extracted the *Symbol IDs* of different nodes under different SNR conditions. We display the results in Figure 9a, where $\left[x_{1},x_{2},x_{3}\right]$ is the *Symbol ID*. Obviously, the *Symbol ID* of one node fluctuates within a certain range under different SNR conditions. However, due to the different deployment locations of each node, the *Symbol ID* of the node can still be distinguished, so the classification method based on the *Symbol ID* is effective. Then we conducted experiments in a realistic environment with a concurrent transmission to test the performance of our scheme in classifying data packets in conflicting scenarios. We placed nodes at different locations in the areas shown in Figure 8c to send packets simultaneously, with the number of nodes set to be 4, 6, and 10. We also adopted the classification algorithm used in CoLoRa to compare with our method. The classification error rates (CERs) are shown in Figure 9b, which shows that our system has a better performance in classification than CoLoRa and performs well even when SNR is as low as −20 dB. We can also find that when SF is larger, the classification performance is greater because the impact of collision symbols in the FFT spectrum is lower, which is consistent with our previous explanation.

### 6.3. Algorithm Performance

**Performance evaluation with an increasing number of LoRa nodes.** We artificially added Gaussian noise to the received signal to control the SNR of the lowest SNR component of the conflicting packets, which is the same as CoLoRa [28] and Nscale [29]. We manually set the SF, bandwidth, and SNR for efficient evaluations. Specifically, the SNR was −20 dB, the SF was 12, and the bandwidth was 125 kHz. Then, we used beacons to synchronize each transmitting node to generate the corresponding number of conflicting packets for evaluation.

**Figure 10:**
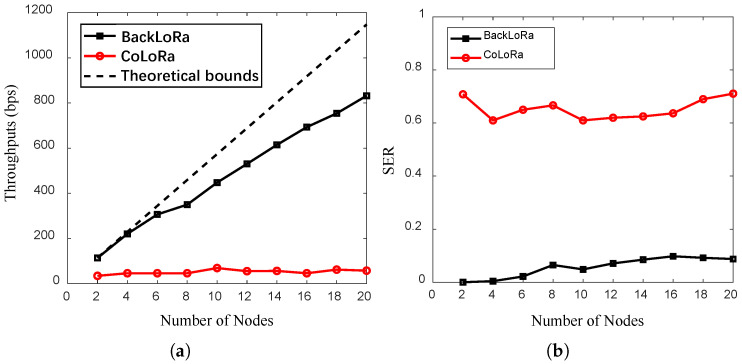
The throughput vs. es (SNR = −20 dB). (**a**) The throughput with an increasing number of nodes. (**b**) The SER with an increasing number of nodes.

As plotted in Figure 10a, the throughput of BackLoRa increased steadily with the number of accumulated nodes. In contrast, the throughput of CoLoRa was always at a low level. For example, the throughput of BackLoRa was about 15× higher than that of CoLoRa when the number of conflict nodes was 20. Furthermore, CoLoRa’s defeat is attributed to three factors: (i) a lack of methods against the distortion caused by noise or interference between adjacent demodulation windows; (ii) the overlapped demodulation peaks of the collided packets, leading to the wrong classifications; and (iii) the fragile SNR (−20 dB), making the receiver fail in detection.

As plotted in the Figure 10b, the SER of BackLoRa was around 10% at SNR = −20 dB when the number of conflicting nodes reached 20. In contrast, the baseline, CoLoRa, was 50% to 70% in these conditions. BackLoRa’s superiority is attributed to parallel demodulation without losing symbol energy, while CoLoRa’s approach, dividing the symbol energy into two random parts based on time bias, reduces the symbol’s energy.

**Figure 11:**
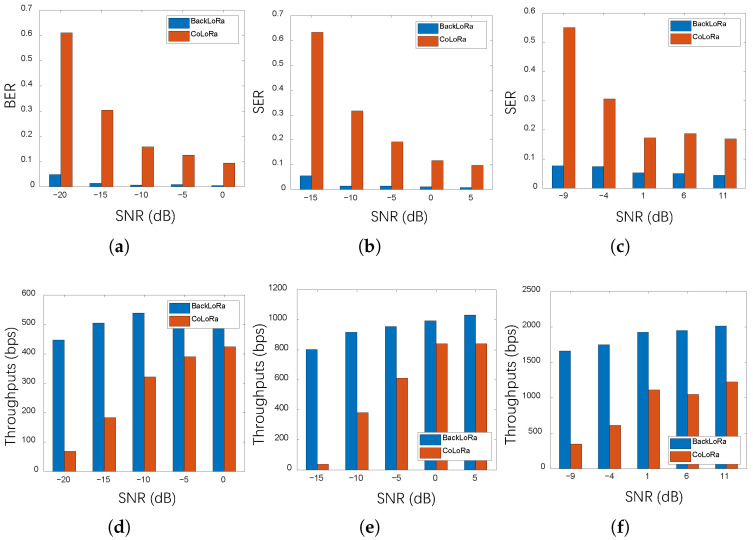
The SER and throughput in different SNRs with changing SF. (**a**) SER performance with different SNRs (SF = 12). (**b**) SER performance with different SNRs (SF = 10). (**c**) SER performance with different SNRs (SF = 8). (**d**) Throughput with different SNRs (SF = 12). (**e**) Throughput with different SNRs (SF = 10). (**f**) Throughput with different SNRs (SF = 8).

Further, we evaluated SF8, SF10, SF12, and SF12 separately with a static LoRa node. Similarly, we added Gaussian noise to the received signal manually as the SNR of the conflicting signal, as shown in Figure 11.

Specifically, as plotted in Figure 11a,d, we evaluated the SER and throughput of BackLoRa in a typical condition in which 10 LoRa nodes collide with every node SF set to 12. The results show that as the SNR increased, both approaches, BackLoRa and CoLoRa, decreased SER and increased throughput. However, BackLoRa performed significantly better when the SNR was low. When the SNR was equal to −20 dB, the SER of BackLoRa was less than 5%, while CoLoRa was higher than 60%. Results demonstrated a 6x performance improvement. Similarly, in Figure 11b,e, which show the SER and throughput, six LoRa nodes collide when SF = 10. Since the bandwidth is 125 kHz when SF = 10, we increased the SNR gradually from −15 dB minimum SNR to ensure a proper data record, with a similar trend. When the SNR was −15 dB, the SER of BackLoRa was about 5%, while the SER of CoLoRa was higher than 60% with extremely low throughput. Similarly, when the SNR reached −15 dB, the BackLoRa could reduce the SER by 57.7% compared to CoLoRa. Moreover, when the SNR was increased to 5 dB, the throughput of BackLoRa improved by about 20% compared to that with −15 dB. Moreover, Figure 11c,f illustrates the SER and throughput of four collided nodes when SF = 8. As the SNR decreased, the superiority of BackLoRa grew significantly. It is worth noting that, in this condition, when the SNR increased to 11 dB, the SER did not drop to a level close to zero. Since both noise and interference between collided packets can cause symbol errors, this phenomenon shows that interference is the main factor for SER reduction.

## 7. Concluding Remarks and Future Directions

This paper presents BackLoRa, a backscatter system that assists with collision-resilient LoRa transmission. We first proposed a hardware and algorithm design to achieve the BackLoRa system. Then, we implemented BackLoRa and conducted comprehensive experiments to evaluate the performance. Evaluation results validate the effectiveness of the BackLoRa system in the real world and indicate that the system can contribute to IoT networks’ usage in future transport areas. However, more in-depth research is needed to achieve this vision. We outline avenues for future research as follows.

- **More parallel transmissions in larger networks.** BackLoRa opens up the possibility of a high-throughput concurrent LoRa network. In the future, we will validate the performance of BackLoRa when LoRa nodes are densely deployed and explore the up-bound of BackLoRa theoretically. Further, we will achieve collision resilience in larger networks by employing the BackLoRa tags’ frequency shift in multiple frequency bands.
- **Lightweight and accurate classification algorithm design.** BackLoRa provides a lightweight and relatively accurate method of collision cancellation, but gaps still exist in the progress towards real-world usage with mass promotion. In the future, BackLoRa can employ some learning-based classification algorithms to ensure light weight and accuracy for real-world usage.
- **Robust design in NLoS conditions.** Our current design achieves collision elimination in line-of-sight (LoS) conditions. However, an interesting research is to achieve robust performance in non-line-of-sight (NLoS) conditions, which can be accomplished by calculating angle of arrival (AoA) with BackLoRa Tags operating as virtual antennas arrays.
- **RF energy harvest and management.** Our current design needs to be powered with a small battery with μW-level power consumption. We can also explore research opportunities for RF energy harvesting and management to achieve battery-free operation.

## Data Availability

Not applicable.
